# Sequencing of the core MHC region of black grouse (*Tetrao tetrix*) and comparative genomics of the galliform MHC

**DOI:** 10.1186/1471-2164-13-553

**Published:** 2012-10-15

**Authors:** Biao Wang, Robert Ekblom, Tanja M Strand, Silvia Portela-Bens, Jacob Höglund

**Affiliations:** 1 Population Biology and Conservation Biology, Department of Ecology and Genetics, Evolutionary Biology Centre, Uppsala University, Norbyvägen 18 D, Uppsala, SE-752 36, Sweden; 2 Evolutionary Biology, Department of Ecology and Genetics, Evolutionary Biology Centre, Uppsala University, Norbyvägen 18 D, Uppsala, SE-752 36, Sweden; 3 Swedish Institute for Communicable Disease Control, Department of Preparedness, Nobels väg, , 18, Solna, SE-171 82, Sweden

## Abstract

**Background:**

The MHC, which is regarded as the most polymorphic region in the genomes of jawed vertebrates, plays a central role in the immune system by encoding various proteins involved in the immune response. The chicken MHC-B genomic region has a highly streamlined gene content compared to mammalian MHCs. Its core region includes genes encoding Class I and Class IIB molecules but is only ~92Kb in length. Sequences of other galliform MHCs show varying degrees of similarity as that of chicken. The black grouse (*Tetrao tetrix*) is a wild galliform bird species which is an important model in conservation genetics and ecology. We sequenced the black grouse core MHC-B region and combined this with available data from related species (chicken, turkey, gold pheasant and quail) to perform a comparative genomics study of the galliform MHC. This kind of analysis has previously been severely hampered by the lack of genomic information on avian MHC regions, and the galliformes is still the only bird lineage where such a comparison is possible.

**Results:**

In this study, we present the complete genomic sequence of the MHC-B locus of black grouse, which is 88,390 bp long and contains 19 genes. It shows the same simplicity as, and almost perfect synteny with, the corresponding genomic region of chicken. We also use 454-transcriptome sequencing to verify expression in 17 of the black grouse MHC-B genes. Multiple sequence inversions of the TAPBP gene and TAP1-TAP2 gene block identify the recombination breakpoints near the BF and BLB genes. Some of the genes in the galliform MHC-B region also seem to have been affected by selective forces, as inferred from deviating phylogenetic signals and elevated rates of non-synonymous nucleotide substitutions.

**Conclusions:**

We conclude that there is large synteny between the MHC-B region of the black grouse and that of other galliform birds, but that some duplications and rearrangements have occurred within this lineage. The MHC-B sequence reported here will provide a valuable resource for future studies on the evolution of the avian MHC genes and on links between immunogenetics and ecology of black grouse.

## Background

The Major Histocompatibility Complex (MHC) plays a central role in the immune system of all jawed vertebrates. It is the most polymorphic genomic region identified, and encodes proteins involved in the innate and adaptive immune responses [1,2]. Particularly, the MHC Class I and Class II genes encode proteins that bind to and carry small antigen peptides to the cell surface thus presenting them to cytotoxic T cells or helper T cells. This in turn triggers the downstream immune cascade. Therefore, this genomic region is crucial for the organism’s resistance and susceptibility to pathogenic disease [2].

Despite its functional consistency, the MHC genomic cluster has different gene organization patterns across different organisms. The latest genomic map of the human MHC (HLA) spans about 7.6 Mb and contains 421 gene loci on a contiguous region on chromosome 6 [3], whereas the MHC regions of other organisms generally have a different gene order and size, or are even scattered on separate chromosomes [4-6]. Notably, the chicken (*Gallus gallus*) has two genetically independent MHC clusters, the MHC-B and MHC-Y (previously Rfp-Y). Both are located on microchromosome 16 (GGA16) [7-11]. There has been some evidence for the gene expression and function for disease susceptibility of the MHC-Y region, but it is the MHC-B that is believed to be the main functional MHC genomic region of chicken [12-15]. The highly streamlined MHC-B, which includes genes encoding Class I and Class IIB molecules, contains only 19 genes and is about 92Kb in length [14-16]. Sequencing efforts have also been made on other bird species, such as mallard duck, red-winged blackbird, house finch and zebra finch [17-21]. However, none of these species seem to share the characteristics of the minimal essential chicken MHC.

The chicken and other fowl species belong to the order Galliformes. Available MHC maps of other galliform birds generally show the same compact feature of this genomic region as that of chicken. For example, the MHC-B of the turkey (*Meleagris gallopavo*) has a good synteny with the chicken MHC-B, the only exceptions being that turkey MHC-B has more BG and BLB (MHC Class IIB) gene copies and an inversion of the TAPBP gene [22]. The quail (*Coturnix japonica*) MHC-B includes an expanded number of duplicated genes and the numbers of the duplicated loci also vary to some extent among individuals [23,24]. The MHC-B of the golden pheasant (*Chrysolophus pictus*) also shows a good synteny with chicken, but has two inversions of TAPBP and TAP1-TAP2 [25].

Black grouse (*Tetrao tetrix*) is a wild galliform bird species that has been well-studied from an ecological perspective, including conservation genetics, behavioural ecology, sexual selection and the evolution of the lek mating system [26-28]. Previous work on the black grouse MHC identified the MHC-B and MHC-Y genomic loci, and the polymorphism of the second exon of the MHC Class IIB gene has been surveyed at the population level [29-31]. In this paper, we investigate the detailed genomic organization of the black grouse MHC-B region. We constructed a fosmid library to sequence the MHC-B genomic cluster and used Roche 454-transcriptome sequencing (RNA-Seq) to verify the expression of the identified genes [32]. The results allow us to conduct a comprehensive comparative genomics analysis of the galliform MHC region. Due to a previous lack of genomic data on avian MHC regions this kind of analysis has not previously been feasible. The black grouse MHC sequence, together with four other completely characterized galliform MHC regions, thus offer a unique opportunity in bird MHC studies.

## Results

### Sequence of the black grouse MHC-B region

**Figure 1:**
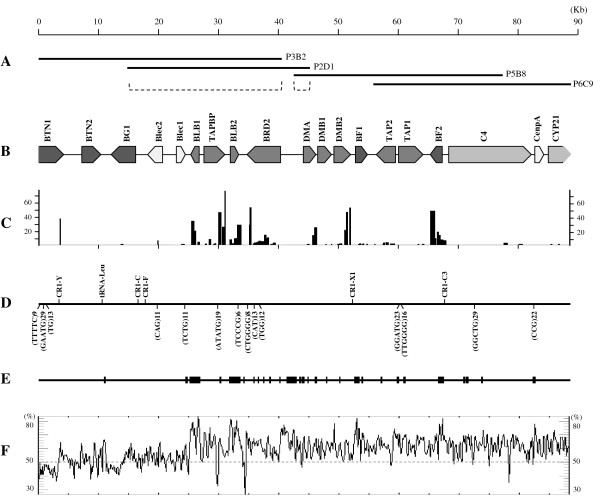
**Sequence features of the black grouse MHC-B region**. **A**. Position of the sequenced fosmid clones. Dotted lines indicate the heterozygous parts. **B**. Gene annotation of the MHC-B of black grouse. Different shadows indicate different MHC gene families defined from human MHC. From dark to light: Class I, Class II, Class III, others. **C**. Average 454 sequencing coverage per nucleotide for each expressed region. **D**. Positions of repetitive elements and tRNAs. **E**. CpG islands in 100 bp window size. **F**. GC contents in 200 bp window size.

Four overlapping MHC-bearing fosmid clones with lengths of 29,972 bp - 40,168 bp were identified and sequenced (Figure 1A). They were aligned into a consensus sequence of 88,390 bp (GenBank accession number JQ028669). This sequence covers the majority of the black grouse MHC-B region (including the complete “core” MHC region), from the BTN1 gene to the CYP21 gene. Since the sequenced black grouse we used was a wild and not inbred animal, we found clones from both homologous chromosomes. More specifically, P2D1 was found to be from a different chromosome than the other three clones (Figure 1A). To maximize the possibility of obtaining a real complete haplotype of the black grouse MHC, we used the combined sequences of P3B2 and P5B8 for the consensus sequence for the heterozygous parts. Therefore, our black grouse MHC sequence was for the most part a real haplotype, apart from the small gap (1,872 bp) between P3B2 and P5B8 which was only covered by P2D1. Sequencing both homologous chromosomes provided us the opportunity to identify polymorphisms in the heterozygous parts. From the heterozygous overlap (25,345 bp) of P3B2 and P2D1, we found 275 single nucleotide polymorphisms (SNPs) and 31 deletion-insertion polymorphisms (DIPs). From the much smaller overlap (2,693 bp) of the P2D1 and P5B8, we found 3 SNPs and 2 DIPs ( Additional file 1).

Five chicken repeats (CR) were identified, of which CR1-F and CR1-X1 were also found to match the chicken MHC-B. We also found 14 simple sequence repeats (SSRs, microsatellites) in the black grouse MHC-B region (Figure 1D, Additional file 2). The average GC content of the black grouse MHC-B region is 59.0%, which is as high as that of the chicken (55.5%) (Figure 1 F). This is probably because the region we sequenced lay on the gene intensive BF/BLB region, which had a higher GC content than the other regions. Also, the black grouse MHC has a high density of CpG islands (Figure 1E), which may indicate the functional importance of this region [33].

### Gene identification and verification

All the three gene prediction programs used could identify most of the genes located on black grouse MHC-B, and most of the chicken, turkey and golden pheasant MHC genes could be well aligned with their homologous genes on black grouse MHC-B. Therefore, 18 genes including BTN1 (partial), BTN2, Blec2, Blec1, BLB1, TAPBP, BLB2, BRD2, DMA, DMB1, DMB2, BF1, TAP2, TAP1, BF2, C4, CenpA, CYP21 (partial) were confirmed at least by three of the above approaches (Table 1). The only exception was the gene BG1: Fgenesh and Genscan did not identify this gene and the comparison with chicken and turkey gave inconsistent results. Therefore, the annotation of this gene is only based on the result from the GeneMark prediction and was checked manually.

**Table 1:** Features of the coding sequences of black grouse MHC-B genes and sequence comparisons with homologous genes in chicken, turkey, quail and pheasant

**Gene**	**Position**	**Strand**	**Gene length (bp)**	**Exons**	Exons verified*	**Average coverage per nucleotide**	**Comparison with other galliformMHCs**								**dN/dS**
							**Chicken**		**Turkey**		**Quail**		**Pheasant**		
							**Nucleotide identity**	**Amino Acid Identity**	**Nucleotide identity**	**Amino Acid Identity**	**Nucleotide identity**	**Amino Acid Identity**	**Nucleotide identity**	**Amino Acid Identity**	
BTN1(partial)	112-4076	+	786	14	1	34.6									
BTN2	7150-10374	+	921	8	0	0	0.797	0.738	0.913	0.894	/	/	/	/	0.470
BG1	12132-16240	-	804	14	1	1.0	0.881	0.826	0.851	0.800	0.832	0.771	/	/	0.335
Blec2	18188-20712	-	825	6	1	5.8	0.698	0.595	0.706	0.593	0.705	0.607	/	/	0.433
Blec1	23007-24547	+	471	5	1	1.0	0.934	0.917	0.949	0.942	0.927	0.894	0.958	0.947	0.230
BLB1**	25386-26738	-	792	6	3	18.1	0.920	0.863	0.932	0.886	0.884	0.826	0.934	0.890	0.805
TAPBP	27591-31078	+	1296	8	7	21.5	0.920	0.882	0.951	0.935	0.891	0.856	0.942	0.919	0.301
BLB2**	32031-33413	+	792	6	5	10.6	0.933	0.897	0.928	0.875	0.890	0.818	0.943	0.902	0.924
BRD2	34798-40437	-	2340	12	12	8.7	0.957	0.996	0.976	0.996	0.934	0.995	0.971	0.997	0.011
DMA	44248-46392	+	789	4	3	12.2	0.913	0.889	0.937	0.924	0.856	0.817	0.943	0.928	0.375
DMB1	46628-48880	+	1020	8	2	1.0	0.864	0.794	0.851	0.793	0.847	0.774	0.923	0.876	0.496
DMB2	49322-52121	+	777	6	5	23.0	0.921	0.911	0.951	0.938	0.859	0.822	0.952	0.955	0.235
BF1***	52919-54907	+	1056	8	5	1.3	0.858	0.769	0.879	0.789	0.835	0.734	0857	0.770	0.733
TAP2	56357-59553	-	2106	9	6	2.2	0.927	0.934	0.952	0.950	0.897	0.893	0.940	0.932	0.186
TAP1	60134-64265	+	1755	11	2	1.0	0.932	0.938	0.957	0.966	0.905	0.915	0.954	0.949	0.195
BF2***	65364-67372	-	1077	8	6	16.1	0.860	0.749	0.888	0.802	0.826	0.737	0.870	0.767	0.767
C4	68440-82244	+	4875	38	4	1.4	0.911	0.940	0.933	0.958	/	/	0.963	0.963	0.207
CenpA	82653-84175	+	429	4	0	0	0.967	0.993	/	/	/	/	0.968	0.979	0.037
CYP21(partial)	84680-88358	+	1326	10	2	1.0									

* Expression verified by 454 transcriptome sequencing, **BLB = MHC Class IIB genes, *** BF = MHC Class I genes.

From our RNA-Seq data, 480 reads could be mapped onto 17 predicted genes in the black grouse MHC-B region, with an average mapped contig length of 209.4 bp. That is, 17 out of the 19 predicted genes (all except BTN2 and CenpA) had concrete evidence of gene expression (Figure 1C). The gene expression levels of the verified genes were variable. For example, BTN1, DMB2 and TAPBP were highly expressed, with mean sequence coverage per nucleotide of 34.6, 23.0 and 21.5, respectively (Table 1). The MHC Class I and Class IIB also had high levels of gene expression. The sequencing coverage per nucleotide of BF2, BLB1 and BLB2 were 16.1, 18.1 and 12.2 respectively. In contrast, the genes BG1, Blec1, DMB1, TAP1 and CYP21 only had one single transcript read mapped each. Within genes, there was a strong 3- prime (including the un-translated region) bias of the number of the transcripts mapped; this is likely due to the technical nature of the cDNA library preparation [34]. The absence of the verification of some exons may also be an artefact of the library preparation, limited sequencing depth or data analysis strategy, and does not necessarily mean that the exons are not expressed [32].

### Comparative genomics of the galliform MHC-B

**Figure 2:**
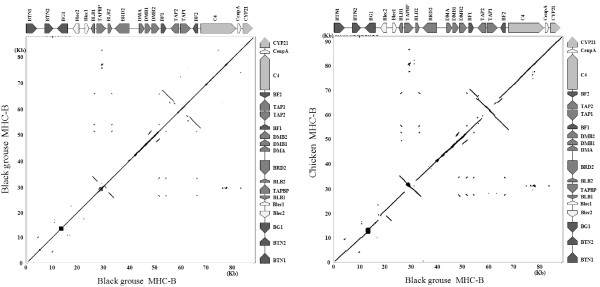
**Identity matrix plotting of the nucleotide sequences of MHC-B region of black grouse itself (left) and between black grouse and chicken (right).** Different shading of genes indicate different MHC gene families defined from human MHC. From dark to light: Class I, Class II, Class III, others.

**Figure 3:**
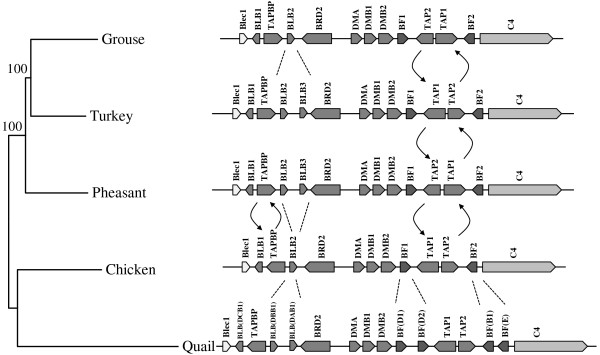
**Phylogenetic relationship and structural comparison of the MHC-B regions of black grouse, chicken, turkey, quail and golden pheasant.** The phylogenetic tree is constructed with the Neighbor-joining method. Numbers next to the branch points indicate the bootstrap values as percentages of 1000 replicates. Pseudogenes of the quail MHC-B are not shown. Arrows and dotted lines highlight inversions and duplications. Numbers beside the arrows indicate the positions of the breakpoints on the compared sequences. Accession numbers: black grouse (JQ028669), chicken (AB268588), turkey (DQ993255), quail (AB078884), golden pheasant (JQ440366). Different shading of genes indicate different MHC gene families defined from human MHC. From dark to light: Class I, Class II, Class III, others.

The black grouse MHC-B genomic region shares an almost perfect synteny with that of chicken, the gene numbers and gene orders of the two species are identical (Figure 2). Compared to the turkey MHC-B, black grouse MHC-B has less BG genes and less BLB genes, but the MHCs of the two species are still highly similar. The golden pheasant MHC-B also has more BLB genes than that of black grouse (Figure 3). The quail MHC-B has significant expansions of BLB genes and BF genes, and has some pseudogenes scattered in this region, but the black grouse MHC-B is still in an obvious synteny with it.

The most remarkable features of the galliform MHC-B is the gene orientation of TAPBP, TAP1 and TAP2. The black grouse MHC-B has inversed TAPBP and TAP1-TAP2 blocks compared to the chicken, while only the TAP1-TAP2 block is inversed compared to the turkey. The golden pheasant shares the same gene orientation of TAPBP and TAP1-TAP2 block with black grouse, where the gene orientation of these gene/gene blocks for quail is the same as that of chicken (Figure 3).

**Figure 4:**
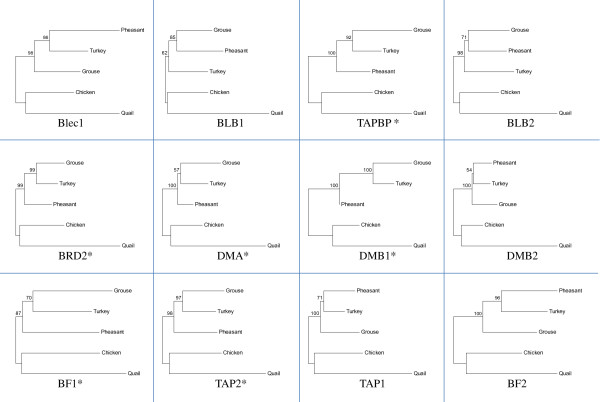
**Phylogenetic relationships of the coding sequences of the homologous genes in black grouse, chicken, turkey, quail and golden pheasant.** The phylogenetic trees are constructed with the Neighbor-joining method. Numbers next to the branch points indicate the bootstrap values as percentages of 1000 replicates. The stars indicate the tree topology is the same as that of neutral makers.

**Figure 5:**
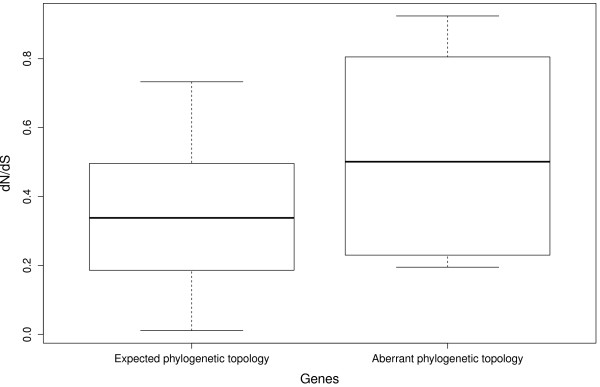
**Plotting of d**_**N**_**/d**_**S**_** values of MHC genes grouped by phylogenetic tree topology.** One group includes the genes following the expected tree topology as neutral markers: TAPBP, BRD2, DMA, DMB1, BF1 and TAP2; the other includes the genes showing aberrant tree topology as neutral markers: Blec1, BLB1, BLB2, DMB2, TAP1 and BF2.

Looking at the genes separately, we found that most of them were very similar in terms of nucleotide and amino acid sequence between the five galliform species (Table 1). However, the phylogenetic relationships of these genes are not consistent. The phylogenetic tree constructed using the entire MHC-B sequences of the five species (Figure 3) follows the neutral expectation [35]. The phylogeny of the coding sequences of TAPBP, BRD2, DMA, DMB1, BF1 and TAP2 share the same tree topology with the tree constructed using the entire MHC-B, whereas the phylogenetic trees for the coding sequences of Blec1, BLB1, BLB2, DMB2, TAP1 and BF2 show different tree topologies within the clade of black grouse, turkey and golden pheasant (Figure 4). Interestingly, genes with aberrant phylogenetic relationships (with grouse or turkey basal to the other two species) showed signs of having elevated d_N_/d_S_ ratios compared to genes following the phylogenetically neutral expectation (Figure 5). This could be interpreted as an indication of increased balancing selection or relaxed purifying selection acting on these genes.

## Discussion

We have sequenced, annotated and analysed the MHC-B gene cluster of the black grouse. Black grouse is a wild bird species and represents the lineage Tetraoninae in the Galliformes [36]. With the availability of its MHC sequence and several other fully sequenced galliform MHC we now, for the first time, have the opportunity to perform a comparative genomic study of avian MHC. The MHC-B gene cluster of black grouse is just as simple and streamlined as that of chicken [15] (Figure 3). By contrast, the quail MHC-B has more duplicated genes and pseudogenes (10 BLB, 7 BF and 8 BG loci) compared to black grouse [23] (Figure 3). The turkey MHC-B and the golden pheasant MHC-B, which are phylogenetically closer to black grouse than chicken and quail, also have expanded BLB genes [22,25] (Figure 3). Our results provide additional evidence that the extremely compact nature of the chicken MHC is not merely an artefact of domestication, since we find a similar pattern in a wild related species that is fully outbred.

The nucleotide identity of the black grouse MHC-B shows high similarity with that of other galliform birds (Table 1). However, individual MHC genes might have different evolutionary histories. The phylogenetic tree based on the entire MHC-B sequence shows exactly the same topology as neutral markers [35] (Figure 3). But when we used the coding sequences of each gene independently, only TAPBP, BRD2, DMA, DMB1, BF1 and TAP2 share the same tree topology with neutral genes (Figure 4). Interestingly, for the genes Blec1, DMB2, TAP1 and BF2, the black grouse is more divergent than turkey and pheasant, while for the two BLB genes (BLB1 and BLB2), black grouse is closer to pheasant than turkey (Figure 4, Additional file 3). If we use the d_N_/d_S_ values to estimate the selection pressure on the genes, we find that the genes following the neutral phylogenetic expectation generally have lower d_N_/d_S_ values than genes with aberrant tree topologies (Figure 5). Taken together the deviation from neutral phylogenetic patterns and elevated d_N_/d_S_ levels indicates that the molecular evolution of several of the genes in the galliform MHC region is affected by selective forces. Especially, the MHC class IIB genes (BLB1 and BLB2) show elevated levels of d_N_/d_S_. The peptide binding regions of these genes are classical examples of balancing selection [37]. An intriguing possibility is that the clustering of the grouse BLB and pheasant BLB might be due to specific selection in the wild since they were both sampled from natural populations, but this hypothesis needs further confirmation.

Another striking finding of the comparison of galliform MHC-B is the repeated inversions of the TAPBP gene and the TAP1-TAP2 block (Figure 3). Using data from all available galliform MHC sequences, we found that the inversion of the TAPBP gene, located between the two MHC class IIB loci, seems to have happened once in the clade; either in the lineage leading to chicken and quail or in the lineage of pheasant, turkey and grouse, depending on the ancestral state. By contrast, the inversion of the TAP1-TAP2 gene block has occurred at least twice (depending on what the ancestral state is, which we cannot tell from our data) during the evolution of this clade. The TAP1-TAP2 block is flanked by the two Class I genes, BF1 and BF2. The events of gene conversion or interlocus recombination in the evolution of MHC genes have been reported before (reviewed in [38]). Here, our result could provide an indirect evidence for such events since if the gene conversion occurred repeatedly, the non-random breakpoints beside the two BF loci may lead to the inversion of the gene block TAP1-TAP2 between them. However, this needs to be further tested.

In this study, we constructed a fosmid library and used it to screen of the MHC genes. Fosmid libraries have been widely used in large genome projects such as gap closure of the human genome or metagenomics analysis [39-41]. The success of our experiment demonstrates that the fosmid library is also suitable and convenient to sequence specific genome regions of a species whose genome map is unavailable. To verify the expression of the identified MHC genes, we mapped the transcriptome data of a 454 sequencing project to the MHC region. This allows us to efficiently confirm the expression of 17 identified genes. However, due to the limited 454 sequencing depth, it was not possible to cover all the 19 putatively expressed genes. Moreover, not all exons were verified in the expressed genes. This could be because of limited sequencing coverage, alternative splicing or artefacts from the mapping method to the short exons [42-44].

## Conclusions

We conclude that there is large synteny between the MHC-B region of the black grouse and that of other galliform birds. Some large scale changes like gene duplications and genomic rearrangements have, however, occurred within the galliform lineage. Some of the genes in the region also seem to have been affected by selective forces within this clade, as inferred from deviating phylogenetic signals and elevated rates of non-synonymous substitutions. The MHC-B sequence of the black grouse reported here will provide a very valuable resource for future studies on the evolution of the avian MHC genes and on immunogenetics and ecology in black grouse.

## Methods

### Genomic sequencing

The genomic DNA used for the sequencing of the MHC cluster in black grouse was extracted from a male bird shot near Östersund, Sweden in November 2009. Muscle tissue was immediately stored in 70% ethanol, -20°C until use. DNA extraction followed the high molecular weight (HMW) protocol described by Blin et al. [45]. The fosmid library was constructed using the Copy Control Fosmid Library Production Kit according to the manufacturer's protocol (Epicentre biotechnology, WI, USA). DNA was first separated by pulsed field gel electrophoresis (PFGE) and 30–39 kb fragments were excised, purified, blunt-ended and ligated into the pCC1FOS fosmid vectors included in the kit. Ligated DNA mixture was then packaged using the supplied lambda packaging extracts and transformed into EPI300-T1 phage *E. coli* hosts. In total the fosmid library consists of approximately 150,000 clones spread over clone pools in twenty 96-well plates.

Screening of the library was performed by a modified PCR-based clone pool method [46]. Nine pairs of PCR primers were used to screen and pinpoint the MHC-bearing clones ( Additional file 4). One of the primer pairs was developed in a previous study of black grouse MHC BLB exon 2 [29], while the others were developed from highly conserved gene regions between Chicken and Turkey. Four overlapping fosmid clones covering the core MHC Class I and Class IIB genes were selected to be sequenced. Shotgun subcloning and Sanger-sequencing of the fosmid clones were performed at 8X coverage by Macrogen (Macrogen Inc., Seoul, Korea). A primer-walking method was used to fill the shotgun sequencing gaps.

The sequencing reads were vector-trimmed, quality-checked and assembled using CAP3 [47]. The assembled fosmid clones were aligned into one consensus sequence using the ClustalW program implemented in CodonCode Aligner 2.06 (CodonCode Corporation, MA, USA) [48]. For the heterozygous parts of overlapping clones, we used the sequences from P3B2 and P5B8 as the consensus sequence (Figure 1A). We also followed a genomic-alignment strategy to detect the putative single nucleotide polymorphisms (SNPs) in the heterozygous parts [49,50]. Alignment of the genomic sequences of the fosmid clones and manual identification of SNPs were conducted using the ClustalW program in CodonCode Aligner 2.06.

### Gene identification

Identification of coding regions and putative exons was conducted by three different gene prediction programs: Fgenesh ( http://www.softberry.com), GeneMark.hmm ( http://exon.gatech.edu) and Genscan ( http://genes.mit.edu/GENSCAN.html) [51-53]. In the Fgenesh and GeneMark.hmm algorithms, the organism-specific parameters were all set as in the chicken; in Genscan, the parameters were set as vertebrate. In addition to the automatic gene identification, we also extracted individual gene sequences from the chicken MHC (GenBank accession number: AB268588 and AL023516), turkey MHC (GenBank accession number: DQ993255) and golden pheasant MHC (GenBank accession number: JQ440366), and used the ClustalW program in CodonCode Aligner to align them with the black grouse sequence to identify the gene positions. Finally, we manually curated the genes by comparing the results from all above approaches, as well as the RNA-Seq mapping result described below. Repeat elements were identified using Repeatmasker ( http://www.repeatmasker.org), and tRNAs were identified using tRNAScan [54]. The identification of CpG islands and the plotting of GC contents were performed using the EMBOSS software suite [55].

### Transcriptome sequencing and gene verification

RNA-Seq data from a 454-transcriptome sequencing project was used to verify expression of the MHC genes (GenBank short read archive number SRA036234) [56]. This data was generated from a male individual collected near Uppsala, Sweden in 2008. Spleen tissue, where many immune-related genes are likely to be expressed, was used to construct the cDNA library. The 454-sequencing was conducted in two partial runs of the GS FLX sequencing instrument (Roche) with Titanium XL reagents and 70x75 mm PicoTiterPlates (PTP). In total 182,179 quality-filtered sequencing reads with average length of 321 ± 141 bp were used for mapping. We used the program gsMapper in Newbler 2.5.3 (Roche/454 Life Sciences) to map the 454-reads to the assembled black grouse MHC consensus sequence. To make sure the mapped reads did not originate from MHC-like paralogues in other genomic regions, we blasted the mapped reads to the entire chicken genome. Reads with a best hit outside the MHC region were excluded in further analysis.

### Comparative genomics analysis

The identity dot matrixes of the black grouse MHC-B sequence and the chicken MHC-B sequence (GenBank accession number: AB268588) were generated using PipMaker [57]. The alignment of the entire MHC-B regions of the five galliform species was performed using the ClustalW program in CodonCode Aligner and the program Mauve 2.3.1 [58] and checked manually. The GenBank accession numbers of the downloaded sequences are AB268588 (chicken), DQ993255 (turkey), JQ440366 (golden pheasant) and AB078884 (quail). The molecular evolution model of the sequences was estimated by jModelTest [59] and the phylogenetic tree was constructed using the neighbor-joining method in MEGA 5.05 [60]. A bootstrap of 1000 replicates was used to verify the creditability of the tree.

The coding sequences of the individual MHC genes were extracted directly from the GenBank entries of the above listed sequences by the GenBank online tools. For the quail, the BF genes beside TAP1-TAP2 block were used as BF1 and BF2 respectively; the BLB genes beside TAPBP gene were used as BLB1 and BLB2 respectively. The alignments of the coding sequences were also conducted using ClustalW in CodonCode Aligner. The phylogenetic trees were constructed following the same protocol as the entire MHC-B tree. The outgroup sequences used to construct phylogenetic trees for pooled BF and pooled BLB genes (in additional file 3) were DQ251182 (domestic goose, *Anser anser*) and DQ490139 (mallard, *Anas platyrhynchos*) respectively. To estimate the molecular selection forces, the rates of nonsynonymous to synonymous (d_N_/d_S_) were calculated using Nei-Gojobori method in the program PAML 4.6 [61,62]. All the pairwise d_N_/d_S_ values between the five galliform species were summarised to calculate the average d_N_/d_S_ value for the gene.

## Competing interests

The authors declare that they have no competing interests.

## Authors’ contributions

BW, TS and JH conceived the study. BW designed the experiments. BW and SP performed the experiments. BW and RE analysed the data and drafted the manuscript. JH supervised all aspects of the study. All the authors read and approved the manuscript.

## Supplementary Material

Additional file 1 Single nucleotide polymorphisms (SNPs) and deletion-insertion polymorphisms (DIPs) identified by comparison of the consensus sequence of black grouse MHC and the sequence of fosmid clone P2D1. Click here for file

Additional file 2 Microsatellites identified from the black grouse MHC sequence. Click here for file

Additional file 3 Phylogenetic trees of pooled BF loci and pooled BLB loci of the five galliform species. Click here for file

Additional file 4 PCR primers used in screening the fosmid library for MHC-bearing clones. Click here for file

## References

[B1] Hughes AL Yeager M Natural selection at major histocompatibility complex loci of vertebrates Annu Rev Genet 1998 32 415 435 10.1146/annurev.genet.32.1.415 9928486

[B2] Klein J Figueroa F Evolution of the major histocompatibility complex CRC Crit Rev Immunol 1986 6 4 295 386 3536303

[B3] Horton R Wilming L Rand V Lovering RC Bruford EA Khodiyar VK Lush MJ Povey S Talbot CC WrighO MW Gene map of the extended human MHC Nat Rev Genet 2004 5 12 889 899 10.1038/nrg1489 15573121

[B4] Kelley J Walter L Trowsdale J Comparative genomics of major histocompatibility complexes Immunogenetics 2005 56 10 683 695 10.1007/s00251-004-0717-7 15605248

[B5] Trowsdale J Both man and bird and beast - comparative organization of Mhc genes Immunogenetics 1995 41 1 1 17 10.1007/BF00188427 7806269

[B6] Kulski JK Shiina T Anzai T Kohara S Inoko H Comparative genomic analysis of the MHC: the evolution of class I duplication blocks, diversity and complexity from shark to man Immunol Rev 2002 190 1 95 122 10.1034/j.1600-065X.2002.19008.x 12493009

[B7] Delany ME Robinson CM Goto RM Miller MM Architecture and organization of chicken microchromosome 16: order of the NOR, MHC-Y, and MHC-B subregions J Hered 2009 100 5 507 514 10.1093/jhered/esp044 19617522

[B8] Solinhac R Leroux S Galkina S Chazara O Feve K Vignoles F Morisson M Derjusheva S Bed'hom B Vignal A Integrative mapping analysis of chicken microchromosome 16 organization BMC Genomics 2010 11 1 616 10.1186/1471-2164-11-616 21050458 PMC3091757

[B9] Fillon V Zoorob R Yerle M Auffray C Vignal A Mapping of the genetically independent chicken major histocompatibility complexes B-@ and RFP-Y-@ to the same microchromosome by two-color fluorescent in situ hybridization Cytogenet Cell Genet 1996 75 1 7 9 10.1159/000134445 8995478

[B10] Miller MM Golo R Bernot A Zoorob R Auffray C Bumstead N Briles WE 2 Mhc class-I and 2 Mhc class-Ii genes map to the chicken Rfp-Y system outside the B-complex Proc Natl Acad Sci USA 1994 91 10 4397 4401 10.1073/pnas.91.10.4397 7910407 PMC43792

[B11] Briles WE Goto RM Auffray C Miller MM A polymorphic system related to but genetically independent of the chicken major histocompatibility complex Immunogenetics 1993 37 6 408 414 8436415 10.1007/BF00222464

[B12] Wakenell PS Miller MM Goto RM Gauderman WJ Briles WE Association between the Rfp-Y haplotype and the incidence of Marek's disease in chickens Immunogenetics 1996 44 4 242 245 10.1007/BF02602552 8753853

[B13] Rogers S Shaw I Ross N Nair V Rothwell L Kaufman J Kaiser P Analysis of part of the chicken Rfp-Y region reveals two novel lectin genes, the first complete genomic sequence of a class I alpha-chain gene, a truncated class II beta-chain gene, and a large CR1 repeat Immunogenetics 2003 55 2 100 108 12692693 10.1007/s00251-003-0553-1

[B14] Kaufman J Volk H Wallny HJ A “minimal essential Mhc” and an “unrecognized Mhc”: two extremes in selection for polymorphism Immunol Rev 1995 143 63 88 10.1111/j.1600-065X.1995.tb00670.x 7558083

[B15] Kaufman J Milne S Gobel TWF Walker BA Jacob JP Auffray C Zoorob R Beck S The chicken B locus is a minimal essential major histocompatibility complex Nature 1999 401 6756 923 925 10.1038/44856 10553909

[B16] Shiina T Briles WE Goto RM Hosomichi K Yanagiya K Shimizu S Inoko H Miller MM Extended gene map reveals tripartite motif, C-type lectin, and Ig superfamily type genes within a subregion of the chicken MHC-B affecting infectious disease J Immunol 2007 178 11 7162 7172 17513765 10.4049/jimmunol.178.11.7162

[B17] Moon DA Veniamin SM Parks-Dely JA Magor KE The MHC of the duck (Anas platyrhynchos) contains five differentially expressed class I genes J Immunol 2005 175 10 6702 6712 16272326 10.4049/jimmunol.175.10.6702

[B18] Edwards SV Gasper J Garrigan D Martindale D Koop BF A 39-kb sequence around a blackbird Mhc class II gene: Ghost of selection past and songbird genome architecture Mol Biol Evol 2000 17 9 1384 1395 10.1093/oxfordjournals.molbev.a026421 10958854

[B19] Hess CM Gasper J Hoekstra HE Hill CE Edwards SV MHC class II pseudogene and genomic signature of a 32-kb cosmid in the house finch (Carpodacus mexicanus) Genome Res 2000 10 5 613 623 10.1101/gr.10.5.613 10810083 PMC310861

[B20] Balakrishnan C Ekblom R Volker M Westerdahl H Godinez R Kotkiewicz H Burt D Graves T Griffin D Warren W Gene duplication and fragmentation in the zebra finch major histocompatibility complex BMC Biol 2010 8 1 29 10.1186/1741-7007-8-29 20359332 PMC2907588

[B21] Ekblom R Stapley J Ball AD Birkhead T Burke T Slate J Genetic mapping of the major histocompatibility complex in the zebra finch (Taeniopygia guttata) Immunogenetics 2011 63 8 523 530 10.1007/s00251-011-0525-9 21494955

[B22] Chaves LD Krueth SB Reed KM Defining the turkey MHC: sequence and genes of the B locus J Immunol 2009 183 10 6530 6537 10.4049/jimmunol.0901310 19864609

[B23] Shiina T Shimizu S Hosomichi K Kohara S Watanabe S Hanzawa K Beck S Kulski JK Inoko H Comparative genomic analysis of two avian (quail and chicken) MHC regions J Immunol 2004 172 11 6751 6763 15153492 10.4049/jimmunol.172.11.6751

[B24] Hosomichi K Shiina T Suzuki S Tanaka M Shimizu S Iwamoto S Hara H Yoshida Y Kulski J Inoko H The major histocompatibility complex (Mhc) class IIB region has greater genomic structural flexibility and diversity in the quail than the chicken BMC Genomics 2006 7 1 322 10.1186/1471-2164-7-322 17184537 PMC1769493

[B25] Ye Q He K Wu SY Wan QH Isolation of a 97-kb minimal essential MHC B locus from a new reverse-4D BAC library of the golden pheasant PLoS One 2012 7 3 e32154 10.1371/journal.pone.0032154 22403630 PMC3293878

[B26] Alatalo RV Hoglund J Lundberg A Lekking in the black grouse - a test of male viability Nature 1991 352 6331 155 156 10.1038/352155a0

[B27] Höglund J Alatalo RV Leks 1995 Princeton: Princeton University Press

[B28] Höglund J Evolutionary conservation genetics 2009 New York: Oxford University Press

[B29] Strand T Westerdahl H Hoeglund J Alatalo RV Siitari H The Mhc class II of the black grouse (tetrao tetrix) consists of low numbers of B and Y genes with variable diversity and expression Immunogenetics 2007 59 9 725 734 10.1007/s00251-007-0234-6 17653538

[B30] Strand T Hoglund J Genotyping of black grouse MHC class II B using reference strand-mediated conformational analysis (RSCA) BMC Res Notes 2011 4 1 183 10.1186/1756-0500-4-183 21672220 PMC3141517

[B31] Strand TM Segelbacher G Quintela M Xiao L Axelsson T Höglund J Can balancing selection on MHC loci counteract genetic drift in small fragmented populations of black grouse? Ecology and Evolution 2012 2 2 341 353 10.1002/ece3.86 22423328 PMC3298947

[B32] Wang Z Gerstein M Snyder M RNA-Seq: a revolutionary tool for transcriptomics Nat Rev Genet 2009 10 1 57 63 10.1038/nrg2484 19015660 PMC2949280

[B33] Antequera F Structure, function and evolution of CpG island promoters Cell Mol Life Sci 2003 60 8 1647 1658 10.1007/s00018-003-3088-6 14504655 PMC11138798

[B34] Ekblom R Balakrishnan C Burke T Slate J Digital gene expression analysis of the zebra finch genome BMC Genomics 2010 11 1 219 10.1186/1471-2164-11-219 20359325 PMC2996964

[B35] Crowe TM Bowie RCK Bloomer P Mandiwana TG Hedderson TAJ Randi E Pereira SL Wakeling J Phylogenetics, biogeography and classification of, and character evolution in, gamebirds (Aves: Galliformes): effects of character exclusion, data partitioning and missing data Cladistics 2006 22 6 495 532 10.1111/j.1096-0031.2006.00120.x 34892896

[B36] Sibley CG Ahlquist JE Phylogeny and classification of the birds of the world 1990 New Haven: Yale University Press

[B37] Hughes AL Nei M Nucleotide substitution at major histocompatibility complex class II loci: evidence for overdominant selection Proc Natl Acad Sci USA 1989 86 3 958 962 10.1073/pnas.86.3.958 2492668 PMC286598

[B38] Martinsohn JT Sousa AB Guethlein LA Howard JC The gene conversion hypothesis of MHC evolution: a review Immunogenetics 1999 50 3–4 168 200 10602879 10.1007/s002510050593

[B39] Bovee D Zhou Y Haugen E Wu Z Hayden HS Gillett W Tuzun E Cooper GM Sampas N Phelps K Closing gaps in the human genome with fosmid resources generated from multiple individuals Nat Genet 2008 40 1 96 101 10.1038/ng.2007.34 18157130

[B40] Kidd JM Cooper GM Donahue WF Hayden HS Sampas N Graves T Hansen N Teague B Alkan C Antonacci F Mapping and sequencing of structural variation from eight human genomes Nature 2008 453 7191 56 64 10.1038/nature06862 18451855 PMC2424287

[B41] Riesenfeld CS Schloss PD Handelsman J Metagenomics: genomic analysis of microbial communities Annu Rev Genet 2004 38 525 552 10.1146/annurev.genet.38.072902.091216 15568985

[B42] Cheung F Haas B Goldberg S May G Xiao Y Town C Sequencing medicago truncatula expressed sequenced tags using 454 life sciences technology BMC Genomics 2006 7 1 272 10.1186/1471-2164-7-272 17062153 PMC1635983

[B43] Emrich SJ Barbazuk WB Li L Schnable PS Gene discovery and annotation using LCM-454 transcriptome sequencing Genome Res 2007 17 1 69 73 17095711 10.1101/gr.5145806 PMC1716268

[B44] Vera JC Wheat CW Fescemyer HW Frilander MJ Crawford DL Hanski I Marden JH Rapid transcriptome characterization for a nonmodel organism using 454 pyrosequencing Mol Ecol 2008 17 7 1636 1647 10.1111/j.1365-294X.2008.03666.x 18266620

[B45] Blin N Stafford DW A general method for isolation of high molecular weight DNA from eukaryotes Nucleic Acids Res 1976 3 9 2303 2308 987581 10.1093/nar/3.9.2303 PMC343085

[B46] Kim CG Fujiyama A Saitou N Construction of a gorilla fosmid library and its PCR screening system Genomics 2003 82 5 571 574 10.1016/S0888-7543(03)00174-5 14559214

[B47] Huang XQ Madan A CAP3: A DNA sequence assembly program Genome Res 1999 9 9 868 877 10.1101/gr.9.9.868 10508846 PMC310812

[B48] Thompson JD Higgins DG Gibson TJ Clustal-W - improving the sensitivity of progressive multiple sequence alignment through sequence weighting, position-specific gap penalties and weight matrix choice Nucleic Acids Res 1994 22 22 4673 4680 10.1093/nar/22.22.4673 7984417 PMC308517

[B49] Taillon-Miller P Gu ZJ Li Q Hillier L Kwok PY Overlapping genomic sequences: a treasure trove of single-nucleotide polymorphisms Genome Res 1998 8 7 748 754 9685323 10.1101/gr.8.7.748 PMC310751

[B50] Mullikin JC Hunt SE Cole CG Mortimore BJ Rice CM Burton J Matthews LH Pavitt R Plumb RW Sims SK An SNP map of human chromosome 22 Nature 2000 407 6803 516 520 10.1038/35035089 11029003

[B51] Burge C Karlin S Prediction of complete gene structures in human genomic DNA J Mol Biol 1997 268 1 78 94 10.1006/jmbi.1997.0951 9149143

[B52] Salamov AA Solovyev VV Ab initio gene finding in Drosophila genomic DNA Genome Res 2000 10 4 516 522 10.1101/gr.10.4.516 10779491 PMC310882

[B53] Borodovsky M Mcininch J Genmark - parallel gene recognition for both DNA strands Comput Chem 1993 17 2 123 133

[B54] Lowe TM Eddy SR tRNAscan-SE: A program for improved detection of transfer RNA genes in genomic sequence Nucleic Acids Res 1997 25 5 955 964 9023104 10.1093/nar/25.5.955 PMC146525

[B55] Rice P Longden I Bleasby A EMBOSS: The European molecular biology open software suite Trends Genet 2000 16 6 276 277 10.1016/S0168-9525(00)02024-2 10827456

[B56] Wang B Ekblom R Castoe TA Jones EP Kozma R Bongcam-Rudloff E Pollock DD Hoglund J Transcriptome sequencing of black grouse (Tetrao tetrix) for immune gene discovery and microsatellite development Open Biol 2012 2 4 120054 10.1098/rsob.120054 22724064 PMC3376728

[B57] Schwartz S Zhang Z Frazer KA Smit A Riemer C Bouck J Gibbs R Hardison R Miller W PipMaker - A Web server for aligning two genomic DNA sequences Genome Res 2000 10 4 577 586 10.1101/gr.10.4.577 10779500 PMC310868

[B58] Darling AC Mau B Blattner FR Perna NT Mauve: multiple alignment of conserved genomic sequence with rearrangements Genome Res 2004 14 7 1394 1403 10.1101/gr.2289704 15231754 PMC442156

[B59] Posada D Crandall KA MODELTEST: testing the model of DNA substitution Bioinformatics 1998 14 9 817 818 10.1093/bioinformatics/14.9.817 9918953

[B60] Tamura K Peterson D Peterson N Stecher G Nei M Kumar S MEGA5: molecular evolutionary genetics analysis using maximum likelihood, evolutionary distance, and maximum parsimony methods Mol Biol Evol 2011 28 10 2731 2739 10.1093/molbev/msr121 21546353 PMC3203626

[B61] Nei M Gojobori T Simple methods for estimating the numbers of synonymous and nonsynonymous nucleotide substitutions Mol Biol Evol 1986 3 5 418 426 3444411 10.1093/oxfordjournals.molbev.a040410

[B62] Yang ZH PAML 4: Phylogenetic analysis by maximum likelihood Mol Biol Evol 2007 24 8 1586 1591 10.1093/molbev/msm088 17483113

