# Supplementary material for: Sequencing of the core MHC region of black grouse (Tetrao tetrix) and comparative genomics of the galliform MHC
Source: BMC Genomics. 2012 Oct 15;13:553. doi: 10.1186/1471-2164-13-553 (PMC3500228; doi:10.1186/1471-2164-13-553)
Supplement: Additional file 1 — Single nucleotide polymorphisms (SNPs) and deletion-insertion polymorphisms (DIPs) identified by comparison of the consensus sequence of black grouse MHC and the sequence of fosmid clone P2D1. [file 1471-2164-13-553-S1.pdf]

Additional file 1. Single nucleotide polymorphisms (SNPs) and deletion-insertion polymorphisms (DIPs) identified by comparison of the consensus sequence of black grouse MHC and the sequence of fosmid clone P2D1.

Position	BGMHC/P2D1
14855	G/A
14898	G/A
14901	A/G
14914	A/G
14915	C/T
14916	A/G
14925	A/T
14941	G/A
14951	C/T
14953	T/C
15036	T/C
15062	A/T
15068	A/G
15069	G/A
15083	G/A
15085	G/C
15090	C/G
15107	C/T
15127	T/G
15133	C/T
15137	T/C
15154	C/G
15157	A/T
15181	C/T
15182	A/G
15185	G/A
15266	G/A
15375	C/A
15403	A/G
15414	G/A
15448	T/C
15449	G/A
15500	T/A
15630	G/A
15638	T/A
15736	T/C
15753	G/T
15846	G/A
15858	A/G
15866	G/A
15899	G/A
15901	C/T
16018	C/A
16146	T/C
16174	G/A
16265	A/G
16274	C/T
16325	T/C

16404	T/C
16438	A/T
16477	A/G
16499	C/T
16638	C/T
16714	A/G
16810	G/A
16848	C/T
16856	C/T
16973	G/T
17011	A/G
17178	A/C
17215	A/G
17334	G/C
17347	A/G
17491	G/A
17581	T/C
17606	A/T
17707	C/T
17866	G/C
17966	C/T
18393	T/G
18494	T/A
18628	T/C
18762	A/C
18988	G/A
19101	C/T
19144	T/C
19185	G/A
19489	A/C
19652	T/A
19786	T/C
20310	T/C
21048	C/A
21217	A/G
21464	C/T
21862	T/C
22074	A/G
22214	G/A
22830	A/G
22927	G/A
22983	A/G
23149	G/A
23235	C/T
23437	G/A
23549	A/G
24790	A/G
24793	C/G
24799	G/T
24894	T/C
25059	T/C
25291	G/A
25314	A/C
25316	C/A

25343	A/G
25472	A/G
25507	C/T
25553	C/A
25558	T/C
25645	T/C
25741	A/G
25986	T/C
26089	G/A
26193	C/A
26195	G/A
26196	A/C
26198	T/C
26218	A/T
26223	G/A
26231	A/C
26239	A/G
26241	G/A
26251	T/C
26252	A/G
26253	T/C
26297	G/C
26338	A/G
26339	T/C
26360	T/C
26363	C/A
26364	C/A
26367	C/G
26372	C/G
26374	T/A
26383	C/T
26413	A/G
26417	T/C
26418	C/A
26419	G/T
26420	C/G
26425	C/A
26527	T/C
26528	G/C
26529	T/C
26531	G/C
26533	C/T
26537	C/A
26555	T/C
26599	T/C
26616	T/G
26709	C/A
26758	T/G
26772	C/T
26774	C/T
26790	G/C
26795	G/A
26877	T/C
26881	T/C

26913	A/G
26914	A/G
26926	C/T
26932	T/C
26935	C/T
26937	T/C
26950	A/T
26972	T/G
26979	C/T
26985	C/G
26992	C/T
26995	T/C
27027	T/G
27046	G/C
27058	T/A
27142	T/C
27159	C/T
27162	T/C
27171	T/G
27198	C/G
27200	G/A
27238	C/T
27255	C/G
27355	T/C
27829	A/G
28090	C/T
28112	C/G
28249	G/A
28476	C/T
28512	T/C
28572	A/C
28584	G/A
28644	C/T
28828	T/C
28980	C/T
29029	T/C
29037	C/T
29038	A/G
29040	A/C
29042	T/C
29044	C/T
29045	A/C
29046	G/A
29048	G/C
29050	C/T
29052	T/C
29055	T/C
29056	G/A
29058	C/A
29060	C/T
29065	A/G
29071	C/T
29074	C/T
29081	T/C

29084 G/A
29095 C/T
29098 T/C
29100 C/T
29101 T/C
29116 C/T
29118 T/C
29119 C/T
29157 A/G
29249 G/A
29509 A/T
29666 A/G
29742 T/A
29821 C/T
29839 A/G
29840 T/G
29841 G/C
29844 A/G
29845 T/A
29846 G/T
29855 T/A
29918 C/T
29939 G/C
29963 A/G
30158 C/T
30192 A/G
30194 A/G
30214 G/A
30549 A/T
31017 A/G
31936 C/A
31964 T/C
31974 T/C
31995 A/G
31997 A/G
32560 T/A
32689 C/T
32792 A/G
33037 T/C
33771 C/T
34543 G/A
35159 G/A
35488 G/A
35882 G/A
35906 T/C
36065 A/G
36318 G/A
36395 G/A
36412 C/T
36842 G/A
36866 C/T
36899 A/G
36911 T/C
36959 C/G

37116	A/C
37144	C/T
37307	G/A
37510	A/G
38287	G/T
38397	G/A
39565	G/A
40066	G/C
40070	G/T
40072	G/A
40080	T/C
42210	A/G
43601	T/C
44748	A/G

Position	BGMHC/P2D1
15676	GAG/---
16123	CGACCTTTGCCCAACTT/-----------------
16486	AT/--
17216	A/-
17607	A/-
17766	ATC/---
17867	CCTGG/-----
22889	AAA/---
23236	-----------/GAGGAAGAGGA
25562	CCCCCTCC/--------
25770	------------/GCTCCCGCTCCC
26944	-/T
27124	AC/--
27960	-/A
28275	C/-
28311	C/-
28836	C/-
29063	C/-
29102	-/A
29120	ACCTCCCCATGTCCCCACA/-------------------
29813	TGA/---
29861	--/AT
33324	GTCCC/-----
33613	GG/--
34413	--/TT
34563	--/AA
35182	CCAGGGCCTCCCCC/--------------
36161	CA/--
39259	-/C
39674	C/-
40067	ACA/---
42334	C/-
42440	CCC/---
