# Supplementary material for: Sequencing of the core MHC region of black grouse (Tetrao tetrix) and comparative genomics of the galliform MHC
Source: BMC Genomics. 2012 Oct 15;13:553. doi: 10.1186/1471-2164-13-553 (PMC3500228; doi:10.1186/1471-2164-13-553)
Supplement: Additional file 2 — Microsatellites identified from the black grouse MHC sequence. [file 1471-2164-13-553-S2.pdf]

Additional file 2. Microsatellites identified from the black grouse MHC sequence.

Motif	Repeat	Start	End
TTTTC	9	81	36
GAATG	29	858	715
TG	13	1178	1153
CAG	11	19833	19799
TCTG	11	24388	24344
ATATG	19	29934	29837
TCCCG	6	33330	33298
CTGGGG	8	34876	34834
CAT	13	36025	35985
TGG	12	36775	36738
GGATG	23	60437	60313
TTGGGG	16	60554	60459
GGCTG	29	72891	72756
CCG	22	82735	82671
