# Supplementary figures and images for: Sequencing of the core MHC region of black grouse (Tetrao tetrix) and comparative genomics of the galliform MHC
Source: BMC Genomics. 2012 Oct 15;13:553. doi: 10.1186/1471-2164-13-553 (PMC3500228; doi:10.1186/1471-2164-13-553)

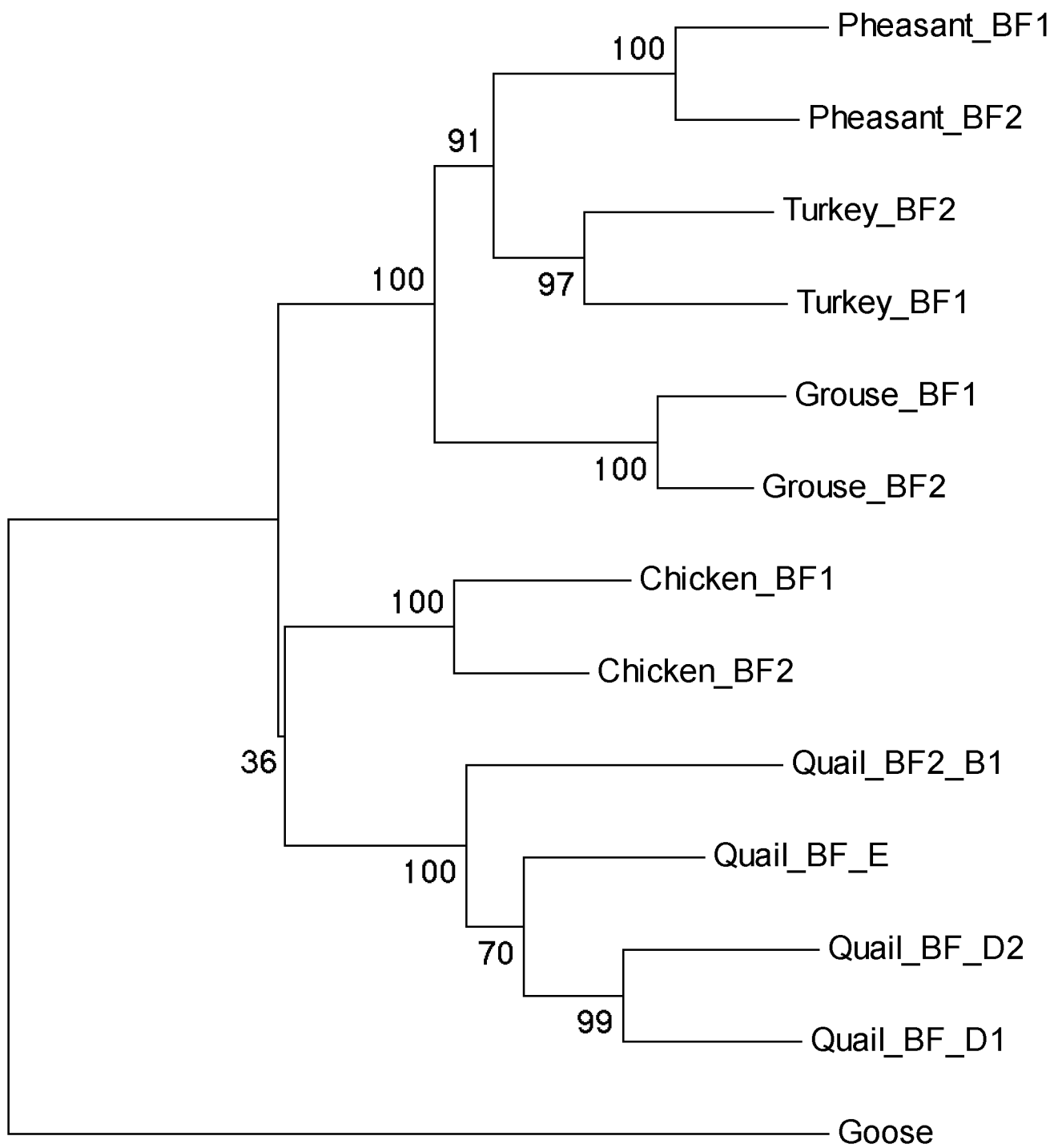

0.05

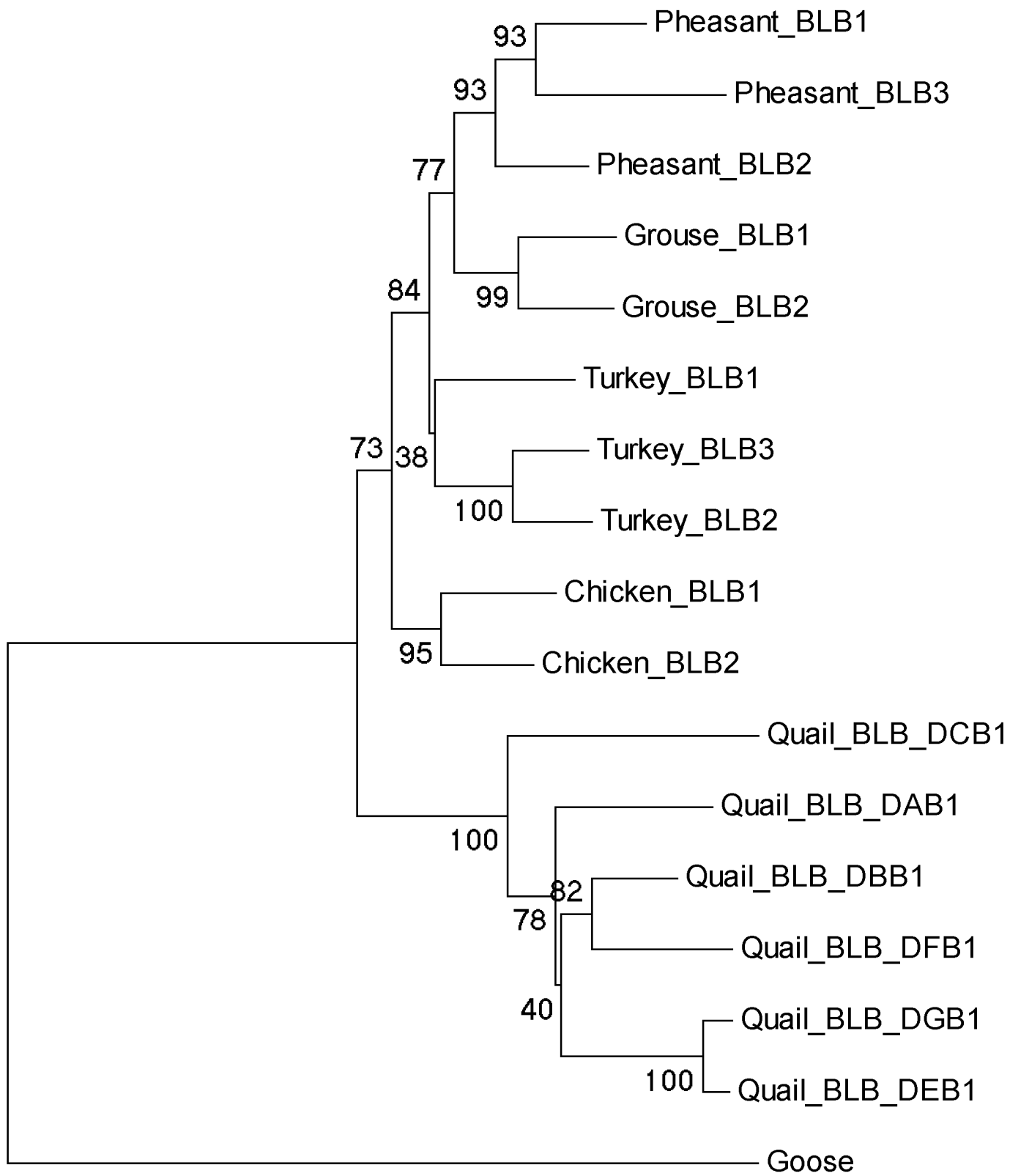

0.05

Supplement: Additional file 3 — Phylogenetic trees of pooled BF loci and pooled BLB loci of the five galliform species. [file 1471-2164-13-553-S3.pdf]
