# Supplementary material for: Sequencing of the core MHC region of black grouse (Tetrao tetrix) and comparative genomics of the galliform MHC
Source: BMC Genomics. 2012 Oct 15;13:553. doi: 10.1186/1471-2164-13-553 (PMC3500228; doi:10.1186/1471-2164-13-553)
Supplement: Additional file 4 — PCR primers used in screening the fosmid library for MHC-bearing clones. [file 1471-2164-13-553-S4.pdf]

Additional file 4. PCR primers used in screening the fosmid library for MHC-bearing clones.

Locus	Sense Primer	Anti-sense Primer	Product Size (bp)
BTN1	TCACCCTGGACCCAAACA	GGAAAGTGAGCCAACCCT	249
BG	ATAGCCATCACCATCTTGC	CTCCGTGCCCTTCTCATT	335
Blec2	TTTCTGGCAGATCCATTCA	GGACAACTCCTCCTTCAACA	203
BLB	GACAGCGAAGTGGGGAAATA	CGCTCCTCTGCACCGTGA	163
BRD	GCTTACGCTTCCTCTTGC	GAGTGGATCGTGCTCTGG	402
DMA	GCGGGTGACAATGCAGGAGT	ACGCTGGTCTGTATGGTGGG	191
BF	CTCTGGTTGAAGCGTTCCTGC	TGCCCTGGTTCGTGTCTGTG	205
TAP1	AGGCCGACAGCAGCGGTGAA	GGACAGCCGTGGTGGCATTG	259
C4	RGTCACCTGGGTCACCTCTG	TCCCTCRGGCTRCRGGAATGG	280
